# Detecting Deception within Small Groups: A Literature Review

**DOI:** 10.3389/fpsyg.2016.01012

**Published:** 2016-06-30

**Authors:** Zarah Vernham, Pär-Anders Granhag, Erik Mac Giolla

**Affiliations:** ^1^Psychology, University of PortsmouthPortsmouth, UK; ^2^Psychology, University of GothenburgGothenburg, Sweden; ^3^Norwegian Police University CollegeOslo, Norway; ^4^Psychology, University of OsloOslo, Norway

**Keywords:** group deception, interviewing, strategies, consistency, memory

## Abstract

Investigators often have multiple suspects to interview in order to determine whether they are guilty or innocent of a crime. Nevertheless, co-offending has been significantly neglected within the deception detection literature. The current review is the first of its kind to discuss co-offending and the importance of examining the detection of deception within groups. Groups of suspects can be interviewed separately (individual interviewing) or simultaneously (collective interviewing) and these differing interviewing styles are assessed throughout the review. The review emphasizes the differences between lone individuals and groups. It focuses on the theoretical implications of group deceit and the reasons why groups need to be understood in terms of investigative interviewing and deception detection if all types of crime-related incidents are to be recognized and dealt with appropriately. Group strategies, consistency within- and between-statements, joint memory, and group dynamics are referred to throughout the review and the importance of developing interview protocols specifically for groups is discussed. The review concludes by identifying the gaps in the literature and suggesting ideas for future research, highlighting that more research is required if we are to obtain a true understanding of the deception occurring within groups and how best to detect it.

Deception detection research has primarily focused on developing an understanding of the strategies that individuals employ when lying or telling the truth about solo crimes, and furthermore what interviewing techniques can be employed by investigators to enable individual liars to be more easily differentiated from individual truth-tellers (e.g., Vrij, 2008; Clemens et al., 2010; Warmelink et al., 2012). However, despite lone individuals being the focus of deception detection research, investigators are often faced with situations whereby they must interview more than one person. Group (or co-) offending (an offense committed by two or more people) is common (McGloin et al., 2008) and on the rise (van Mastrigt and Farrington, 2011). Studies suggest that co-offending accounts for between 10 and 17% of police recorded offenses with between 22 and 30% of offenders being involved in co-offending (Carrington, 2002; Hodgson, 2007; Van Mastrigt and Farrington, 2009; D’Alessio and Stolzenberg, 2010; Ouellet et al., 2013). However, co-offending statistics need to be interpreted with caution. This is because the data available tends to only represent those offenders who have been charged or detected, resulting in a sizeable dark figure of co-offending (e.g., a crime may be recorded as a solo event when in fact investigators are just unaware that more offenders were involved).

Co-offending is just one circumstance that would require the investigation of more than one person. Other contexts in which multiple individuals may need to be questioned are when there are multiple eyewitnesses to a specific crime or when a suspect reports an alibi witness (someone who can provide an account of the whereabouts of a suspect at a location other than the crime scene at the time the crime took place; Dahl and Price, 2012). Additionally, some criminal groups may consist of members who are involved only in the planning, or in the aftermath, of an offense, but not in the actual commission of the offense itself. Similar to alibi witnesses, these group members will be collaborators of the crime but not active participants. Despite having different group dynamics to multiple suspects, there is still the potential for investigators to question more than one person about the same crime/event. However, in these contexts the aim for investigators is to determine the accuracy of the information provided by the eyewitnesses, to determine whether the alibi witness is true or false, or to determine the differing roles played by the group members in the offense process.

Although investigators often have to question groups, whether it is multiple suspects, multiple eyewitnesses, or alibi witness situations, the number of deception detection studies involving groups is limited. Our literature search found only 20 studies published on the topic of verbal group deception (see **Table 1**). All 20 studies, except one, considered multiple suspects; therefore it is groups of suspects that will be the primary focus of the current review. The aim of the current review article is to collate and discuss the research that has been conducted into interviewing groups to detect deceit and to discuss the theoretical implications of this upcoming research area. The review will consider the differences between groups and individuals, and will report the different aspects of the extant group deception studies, for example, interviewing group members separately vs. interviewing group members simultaneously. Throughout, the review gaps in the literature will be identified with suggestions for future studies. The current review will focus on the verbal indicators of deceit and therefore studies measuring physiological measures to detect deceit in groups will not be discussed (see Bradley and Barefoot, 2010; Meijer et al., 2010, 2013, for studies using the Concealed Information Test (CIT) to extract information from groups).

**Table 1:** Overview of the group deception studies completed so far, which measure verbal cues (spanning across 11 years from 2003 to 2014).

Group deception study	Theoretical principle examined	Interview style	Study manipulation	Number of interviewees	Adults or children?	Published?
Granhag et al., 2013	Strategies	Individual	SUE technique	3	Adults	Yes
Vrij et al., 2010c	Strategies	Individual	(Un)anticipated questions	2	Adults	Yes
Chan and Bull, 2014	Consistency	Individual	Effect of co-planning	2	Adults	Yes
Granhag et al., 2014	Consistency	Individual	SUE technique	3	Adults	Yes
Granhag et al., 2003	Consistency	Individual	Repeated interviews	2	Adults	Yes
Leins et al., 2011	Consistency	Individual	Drawings	2	Adults	Yes
Mac Giolla and Granhag, 2015	Consistency	Individual	(Un)anticipated questions: intentions	3	Adults	Yes
Roos af Hjelmsäter et al., 2014	Consistency	Individual	Sketches	3	Children	Yes
Sooniste et al., 2014	Consistency	Individual	(Un)anticipated questions: intentions	2 and 4	Adults	Yes
Strömwall and Granhag, 2007	Consistency	Individual	Response mode of observers	2	Children	Yes
Strömwall et al., 2003	Consistency	Individual	Repeated interviews	2	Adults	Yes
Vredeveldt and Wagenaar, 2013	Consistency	Individual	(Un)anticipated questions	2	Children	Yes
Vrij et al., 2009	Consistency	Individual	(Un)anticipated questions	2	Adults	Yes
Driskell et al., 2012	Memory	Collective	Brief investigative interview	2	Adults	Yes
Jundi et al., 2013a	Memory	Collective	Timeline task	2	Adults	Yes
Jundi et al., 2013b	Memory	Collective	Pairs’ monitoring of the interviewer	2	Adults	Yes
Vernham et al., 2014a	Memory	Collective	(Un)anticipated questions	2	Adults	Yes
Vrij et al., 2012	Memory	Collective	(Un)anticipated questions	2	Adults	Yes
Vernham et al., 2014b	Imposing cognitive load	Collective	Forced turn-taking technique	2	Adults	Yes
Nahari and Vrij, 2014^∗^	Verifiability approach	Collective	Written statements	2	Adults	Yes


*SUE, Strategic Use of Evidence.*

*^∗^This study involved alibi witness scenarios as opposed to multiple suspects.*

## Defining A Group

A group can be defined as “two or more individuals who are connected to one another by social relationships” (Forsyth, 2006, p. 3). Group deception studies, to date, have primarily focused on dyads, and the majority of group offenses in real-life involve dyads (Hodgson and Costello, 2006). Nevertheless, it is important to note that there is much debate within the group dynamics literature with regard to the definition of a ‘group’ and whether a dyad actually constitutes a group (often referred to as the Moreland-Williams debate). Levine and Moreland (2012) argue that dyads are not groups because the number of individuals within the group matters. That is, larger groups involve different group processes. They state that dyads are different from larger groups because; (1) they form and dissolve more quickly; (2) they are more emotionally involved with one another; and (3) certain social phenomena, such as minority/majority relations, cannot be applied unless there is more than two people in the group. In contrast, Williams (2010) states that dyads should come under the definition of a group because group processes, such as social loafing and facilitation or ingroup/outgroup, occur regardless of whether there are two or several people in the group. Consequently, dyads are appropriate targets for investigation. Williams (2010) reports that it is the scientific methods employed which are important to ensure that reliable data is collected about how individuals behave when around others. If dyads do not constitute a group, nor are they individuals, then dyads will need to be studied as a field on their own. As a result of this controversial debate within the group dynamics literature, it is important that group size is considered when interpreting the findings and forming conclusions from the current review. Nevertheless, the fact that dyads are different from individuals regardless of whether they come under the term ‘group,’ means that the current review is still necessary and relevant for those involved in investigative interviewing and detecting deception. For the purpose of the current review and in line with the definition by Forsyth (2006) mentioned above, dyads will be thought of as a group.

## Group Deception: How Are Groups Different From Individuals?

Although the knowledge-base surrounding detecting deceit in groups is limited, it is important to acknowledge that groups are different from individuals (e.g., in terms of shared responsibility and peer support; Warr, 2002). Consequently, there are numerous reasons why it is important to expand the investigative interviewing and deception detection literature to include groups. First, the characteristics of group offenders differ from the characteristics of solo offenders. For example, co-offenders are typically younger than solo offenders (Van Mastrigt and Farrington, 2009). Furthermore, it seems that groups are more strategic and view honesty and deception differently from individuals. Groups, for instance, lie more than individuals (Cohen et al., 2009) and are more likely to use honesty strategically in order to maximize their own outcomes (e.g., financial payoffs; Sutter, 2009). Groups also report more self-interest (both collective self-interest and individual self-interest) and fewer concerns about using deception in comparison to individuals (Cohen et al., 2009). These differences in motives, strategies and behaviors highlight the need to understand group deception as well as individual deception. Additionally, there are specific crimes (e.g., organized crime, terrorism, drug trafficking, burglary, arson) and multiple investigative settings (e.g., immigration, airport security, border control, police stop and search) that are more likely to involve groups of offenders as opposed to lone offenders (Carrington, 2002; Van Mastrigt and Farrington, 2009).

Second, the interviewing of groups brings a different dynamic to the interview process. This allows for different interview approaches to be applied and enables the identification of unique cues to deceit that can only emerge when groups are interviewed. A central issue that investigators face is whether to interview groups individually (i.e., interviewing of group members separately) or collectively (i.e., interviewing of group members simultaneously). Interviewing group members individually provides the potential to examine within-group consistency (Strömwall et al., 2003; Granhag et al., 2014), which cannot be measured when interviewing a lone individual about a solo crime. Additionally, collective interviewing allows for communication and interaction cues to be examined (Driskell et al., 2012; Vrij et al., 2012; Jundi et al., 2013b; Vernham et al., 2014b), and these cues cannot emerge when interviewing a lone individual about a solo crime or when individually interviewing group members about a joint crime. Currently, police typically conduct individual interviews during their investigations regardless of the number of suspects. Nonetheless, there are some existing procedures where collective interviewing is employed. For example, in the UK, immigration officers occasionally use collective interviewing when attempting to uncover sham marriages (Home Office, 2013), and police detectives often interview people in groups when making house-to-house enquiries (College of Policing, 2013). In Canada, customs officers carry out collective interviews at airports because members of the same group are deemed to have a ‘similar agenda’ (i.e., it is assumed that group members traveling together are traveling to the same destination for the same purpose). Thus, if only one person in the group is examined, this could result in a wasted effort or missed opportunity. That is, the interviewing of group members individually will be less time-efficient and may result in specific individuals who need to be interviewed being disregarded (personal communication with a Canadian ex-immigration officer, 12th November 2013). By extending the research agenda to include collective interviewing, research can inform on the best practices for such situations and has the potential to uncover new applied contexts where collective interviewing may be appropriate.

Third, for groups you can determine deception at a social level as well as at an individual level. In other words, interviewing groups of suspects not only allows for the additional consistency and social cues (as mentioned above) to be measured, but also allows for the measurement of established deception cues within each individual of the group (e.g., within-statement consistency, number of details, admitting lack of memory, plausibility, spontaneous corrections; Granhag and Strömwall, 1999; DePaulo et al., 2003; Vrij, 2005, 2008). Therefore, by examining individual deception and group deception more cues to deceit can emerge from interviewing group members about joint events than from interviewing lone individuals about solo events. The identification of both known and novel cues to deceit from groups is important if we are to improve the poor accuracy rates typically found within the detection deception literature (Bond and DePaulo, 2006) and obtain knowledge of what cues are best to focus on in each given investigative situation.

Fourth, when multiple people need to be questioned, each member of the group loses some control over what information is provided and how each member behaves. Therefore, not only do they have to ‘impression manage’ themselves (i.e., regulate and control what information they say), but they also have to ‘impression manage’ others (i.e., attempt to regulate, predict, and control what others say). This suggests that the strategies employed by groups will differ from the strategies employed by lone individuals because group members will not be as successful as lone individuals when improvising on the spot during the interview. This will be the case regardless of whether the group members are interviewed individually or collectively. The notion of impression management can be linked to research examining the ‘Prisoner’s Dilemma’ (Rapoport and Chammah, 1965; Poundstone, 1992), especially when group members are interviewed separately about a crime. That is, each member of the group will need to make a decision about whether they can trust the other group members to cooperate with the group and not the police. Such reasoning is difficult. Therefore the added task of impression managing others implies that group situations are naturally more cognitively demanding: not only do you have to think about and remember what you have said but you also need to consider what others have said or might be saying. Research shows that increased cognitive load results in more cues to deceit (Vrij et al., 2008, 2010b). Hence, cues to deceit may not only be more numerous but also strengthened in group situations.

In order for law enforcement to be competent in dealing with all forms of crime-related incidents, they need to be trained in a variety of interview techniques that enable them to question both individuals and groups. Different situations may require different interviewing techniques. It is therefore important that investigators have a wide range of all the necessary skills to be able to use the most appropriate interview strategy for the situation at hand, and to be able to recognize the cues indicative of deceit that emerge from implementing that particular interview technique. For example, if there is only one interviewer available but multiple suspects, employing a collective interviewing approach would be most convenient and time-efficient. When this approach is implemented, the focus should be on cues associated with how the interviewees interact and communicate with one another, such as verbal transitions (Driskell et al., 2012) or eye contact (Jundi et al., 2013b).

## Theoretical Implications of Detecting Deception Within Groups: What Do the Existing Deception Studies Tell Us?

From a theoretical perspective, research on the interviewing of groups contributes to the knowledge-base within the deception literature. It adds to the consistency research (e.g., repeat vs. reconstructive hypothesis; Granhag and Strömwall, 1999), compliments memory research (e.g., collaborative learning and remembering), and can be applied to the theories of co-offending (e.g., social exchange theory; Weerman, 2003) and other group processes (e.g., group formation and leadership). Hence, the interviewing of groups about joint events not only allows for the development of new interview techniques that increase information elicitation (crucial to detecting deceit), but also allows for additional theories and concepts to be applied to deception that cannot be applied when interviewing lone individuals about solo events. In the following sections, we will review the existing deception research on group interviewing which has contributed to the literature on; counter-interrogation strategies (e.g., Granhag et al., 2013), within-group consistency (e.g., Strömwall et al., 2003), transactive memory (e.g., Wegner, 1987), and collective memory (e.g., Barnier and Sutton, 2008). We will also highlight theoretical positions we feel are of utmost relevance to group deception, but have yet to be empirically examined, such as group dynamics (e.g., Arrow et al., 2000).

### Strategies

Knowledge about the strategies that truth-tellers and liars employ is important if investigators are to have insight into what truth-tellers and liars will say, and how they will behave, during an interview. If this is known, more insight can be acquired with regard to the deception cues that may arise, and most importantly, more effective and theory-based interview protocols can be developed to help improve investigators’ abilities to accurately detect deceit (see Granhag et al., 2015 for an overview). Despite this, most research has focused on the verbal and non-verbal behaviors of truth-tellers and liars, with little research being conducted into the actual strategies that truth-tellers and liars employ to appear credible (e.g., DePaulo et al., 2003; Vrij, 2008). This is particularly the case for the strategies employed by truth-telling groups and lying groups (see Vrij et al., 2010c; Granhag et al., 2013 for the only two deception studies to examine group strategies when individual interviewing is applied).

When faced with the situation of being questioned about their activities or whereabouts at the time of a crime, truth-tellers and liars (whether an individual or part of a group) have the same goal and that is to convince the interviewer of their innocence (Granhag and Hartwig, 2008). Research into the differing strategies employed by truth-telling groups and lying groups, when group members are interviewed individually, demonstrates that although there are no differences in terms of the non-verbal strategies (e.g., both truth-telling groups and lying groups plan to suppress nervous behaviors), there are key differences in terms of the verbal strategies they use (i.e., how they provide information; Vrij et al., 2010c). This is also the case for the non-verbal and verbal strategies used by lone individuals (Strömwall et al., 2006).

Truth-telling groups are less likely to have a strategy than lying groups because truth-tellers believe in a ‘just world’ (Lerner, 1980) and that the truth will shine through (‘illusion of transparency’; Gilovich et al., 1998). Consequently, they rely on memory, as opposed to preparation, to provide their answers. Truth-telling groups prefer a ‘tell it all’ strategy that aims to provide an honest and detailed description of what actually occurred (Vrij et al., 2010c; Granhag et al., 2013). Even when part of a group, truth-tellers do not feel the need to prepare, believing their statements will naturally be consistent with one another. The only time truth-telling groups may prepare is to run through what happened in order to remind one another of the details of the event (Vrij et al., 2010c).

Conversely, lying groups, who are less likely to take their credibility for granted, are more likely to prepare for an interview. They plan what to say beforehand and prepare fabricated stories that are coherent and plausible. Lying groups prepare joint alibis, preferring a ‘keep it simple’ strategy in order to avoid raising suspicion and to ensure consistency within and between their statements (Vrij et al., 2010c; Granhag et al., 2013). They will prepare answers to possible questions to ‘get their stories straight,’ but their answers will be restrictive and vague to reduce the chances of them contradicting one another or providing incriminating evidence (Vrij et al., 2010c; Granhag et al., 2013). These differing strategies employed by groups of truth-tellers and groups of liars results in the statements from lying groups being less detailed than the statements from truth-telling groups. However, because lying groups have planned what to say together, their statements are often as consistent as the statements of truth-telling groups (Granhag et al., 2003; Chan and Bull, 2014).

As a result of the strategies that lying groups employ, opportunity for planning is likely to be an important moderator of credibility. Research on lone individuals has shown that liars who prepare for an interview are more difficult to distinguish from truth-tellers, than liars who do not prepare (O’Hair et al., 1981; Bond and DePaulo, 2006). Preparing what to say in an interview enhances liars’ deceptive performance (Granhag et al., 2004) by making their statements not only more consistent than truth-tellers’ (Granhag et al., 2003), but also more immediate and plausible (Chan and Bull, 2014). Consequently, the emergence of cues to deceit is reduced (DePaulo et al., 2003). Nevertheless, the influence of opportunity to plan has not been empirically tested in groups of interviewees, and therefore studies examining the effects of planning on lie catchers’ abilities to distinguish between groups of truth-tellers and groups of liars are required.

The Al Qaeda Handbook (a terrorist training manual found in 2000 by Manchester police in England) offers further insights into the strategies of groups of suspects. Amongst other things, this handbook underscores the importance of a group security plan that all group members are to commit to. This plan emphasizes the importance of teamwork and explains how to undertake group missions, which includes practicing answers to a list of anticipated interview questions. Additionally, it covers counter-interrogation strategies. Of specific relevance to group interviewing situations are the strategies that state; (i) always stick to the cover story even when shown evidence of involvement, and (ii) if interrogators state that other group members have revealed information, just agree, but do not state any additional information. This manual therefore gives insight into the types of strategies that groups may employ in order to; (1) co-ordinate with other group members; (2) avoid revealing incriminating information; and (3) avoid deviating from the group security plan. However, it assumes that group members will be interviewed separately. Therefore, interviewing group members collectively may be an interview tactic that groups typically do not prepare for, which may increase cues to deceit.

To summarize, the fact that research shows that groups of truth-tellers and groups of liars use the same non-verbal strategies but different verbal strategies helps to explain why verbal cues are deemed more diagnostic to deceit, than non-verbal cues (which is the case for both adults and children; Strömwall and Granhag, 2007; Vrij, 2008). Furthermore, whether a lone individual or part of a group, the strategies employed seem to be very similar. However, there are likely to be key differences with regard to the strategies employed by truth-tellers and liars depending on whether they are part of a group (as suggested by the Al Qaeda Handbook), because groups will need to decide what to do if the planned strategies cannot be employed during the interview. More studies investigating the strategies that groups of truth-tellers and groups of liars employ are required. The few deception studies that have considered strategies have involved interviewing groups consisting of two or three members separately. This means that the strategies employed by larger groups have been ignored, as have the strategies employed by groups interviewed collectively. Group size is unlikely to influence the counter-interrogation strategies employed, because the theoretical principles of the strategies remain the same. However, the outcome of implementing the strategies may differ as group size increases, because the ability to remain consistent will become more challenging. Future studies will need to examine this before any conclusions can be drawn. Collective interviewing raises further questions with regard to suspect strategies. For instance, will group strategies cover the communication and interaction cues that are available during collective interviewing? Or how do group members react and alter their strategies when informed that one group member has provided information other than that agreed upon in the pre-planned story? Future research on group strategies should address such questions.

### Consistency

Verbal consistency is regarded by many as an important cue to deceit. Both laymen (The Global Deception Research Team, 2006) and professionals (Akehurst et al., 1996; Strömwall and Granhag, 2003) assume that inconsistency is indicative of deceit. However, it is often overlooked that consistency comes in many forms. Single statements can be examined for within-statement consistency (the level of consistency within one statement from an individual), while repeated statements can be examined for between-statement consistency (the level of consistency between multiple statements from one individual). When groups of individuals are interviewed seperately an additional form of consistency emerges: within-group consistency (the level of consistency between statements from group members; see Vredeveldt et al., 2014, for an overview of consistency cues in deception contexts). The first studies on group deception began with this cue in mind (Granhag et al., 2003; Strömwall et al., 2003), and, considering the importance that people place on consistency as a cue to deceit, it is perhaps not surprising that the majority of studies on group deception have continued to focus on this cue (see **Table 1**). However, it is important to note that all the group deception studies that have considered consistency as a cue to deceit have involved interviewing the group members individually. Future studies should consider measuring consistency as a possible cue to deceit in collective interviewing situations.

If the strategies that truth-telling groups and lying groups employ are taken into consideration (i.e., truth-tellers rely on memory whereas liars plan what to say), it may be problematic to assume that truth-tellers are more consistent than liars. Accordingly, the consistency of truth-telling groups is often equal to (or even weaker) than the consistency of lying groups (e.g., Granhag et al., 2003; Strömwall et al., 2003). To understand this further, Granhag and Strömwall (1999) proposed the ‘repeat vs. reconstruct’ hypothesis, which emphasizes that liars will attempt to *repeat* what they have previously said and truth-tellers will try to *reconstruct* what they actually experienced. When truth-tellers are asked to repeat answers, their memory restructures the event so they gain, lose, and change information over time (Baddeley, 1990), thus reducing consistency. In contrast, liars merely repeat what they originally prepared, thus promoting consistency. Although, the ‘repeat vs. reconstruct’ hypothesis was originally developed to measure the consistency between two statements from the same interviewees (Granhag and Strömwall, 1999), later research has found that this hypothesis can also be applied when measuring the consistency between statements of multiple interviewees (e.g., Granhag et al., 2003). Research suggests that there is a variation in the types of details that groups provide, with truth-telling groups focusing more on the salient aspects of an event than lying groups. Thus, when these salient aspects are compared, consistency is significantly greater for truth-telling groups compared with lying groups (Roos af Hjelmsäter et al., 2014). Future research should consider whether there are specific types of details that truth-tellers reconstruct and liars repeat (e.g., salient/central details vs. non-salient/general details).

Relevant to the ‘repeat vs. reconstruct’ hypothesis is the ‘reminiscence effect’ which suggests that repeated questioning results in the recall of previously unrecalled items (referred to as commission errors; Payne, 1987). This effect has been found to be stronger for truthful statements than for deceptive statements (Granhag et al., 2003). Therefore, in contradiction to the stereotypical belief that consistency implies truthfulness (see ‘consistency heuristic’ literature; Granhag and Strömwall, 2000), it is clear that lie catchers need to be cautious when interpreting consistent statements as truthful and inconsistent statements as deceitful. In fact, the diagnostic value (i.e., predictive accuracy) of using consistency to judge veracity in groups is modest for both adults (Strömwall et al., 2003 obtained overall accuracy rates of between 52.5 and 70%) and children (Strömwall and Granhag, 2007 obtained an overall accuracy rate of 62.5%). These modest accuracy rates are not only because lie catchers are exercising the consistency heuristic incorrectly, but also because judging consistency is a subjective task (i.e., different observers can perceive the same set of statements differently in terms of consistency; Granhag and Strömwall, 2000).

The diagnostic value of the consistency cue can vary depending on the response mode used. The response mode refers to the stage at which a lie catcher makes a veracity judgment, and this can vary when you have repeated interviewing and/or groups of interviewees. Research implies that observers are more accurate at using the consistency cue when they use a step-by-step response mode (observers make a veracity judgment after seeing each interrogation) compared to when they use an end-of-sequence response mode (observers make one veracity judgment after seeing all interrogations with all group members). This is because the step-by-step response mode facilitates more effective information processing of inconsistencies (Strömwall et al., 2003; Strömwall and Granhag, 2007). Therefore, the diagnostic value of the consistency cue improves because there is a reduction in the degree of truth bias (the predisposition for observers to judge someone as telling the truth; Street and Masip, 2015). However, the differences in accuracy rates depending on response mode vary across the few studies available and sometimes only approach significance (e.g., Strömwall et al., 2003); hence, more research is needed into the effects of response mode on the accuracy rates of detecting deceit in groups before any conclusions can be made.

An important development in deception research in recent years is the introduction of strategic interviewing techniques (Vrij and Granhag, 2012). These techniques involve asking interview questions that play on the differing strategies of truth-tellers and liars. The most relevant method applied to within-group consistency is simply to ask unanticipated interview questions, which negate the benefit of planning for the interview (Vrij et al., 2009; Lancaster et al., 2012). Unanticipated interview questions are designed to disrupt liars’ repeat strategy, thereby reducing within-group consistency. If framed correctly truth-tellers’ consistency levels are unaffected because truth-tellers can still rely on memory to answer such questions. For example, Vrij et al. (2009) found that, when pairs were interviewed individually, there was less agreement between the answers from lying pairs, compared to truth-telling pairs, but only for unanticipated interview questions, such as questions concerning spatial details (accuracy rates for truth-tellers and liars ranged from 60 to 80%). When anticipated interview questions were asked, no differences were found between truth-telling pairs and lying pairs. Further studies have supported these findings with adult groups (Leins et al., 2011), child groups (Vredeveldt and Wagenaar, 2013; Roos af Hjelmsäter et al., 2014), and when the statements are on true and false intentions, as opposed to past events (Sooniste et al., 2014; but see Mac Giolla and Granhag, 2015 where truth-tellers showed higher levels of consistency for both anticipated and unanticipated questions). The accuracy rates obtained for truth-tellers and liars for the within-group studies that measure the classification of participants based on veracity are impressive, ranging from approximately 60 to 100%. These studies emphasize the need for investigators to develop interview protocols that include both expected and unexpected questions if they are to improve the diagnostic value of the consistency cue and enhance the accuracy rates of lie catchers whom are detecting deceit in groups.

When specific interview techniques are employed, such as the Strategic Use of Evidence (SUE) technique (Hartwig et al., 2006), a further consistency cue can be measured in addition to those already mentioned: statement-evidence consistency (the degree of consistency between the suspects statements and the evidence that the interrogator holds). Granhag et al. (2014) illustrated that when the SUE technique was employed during individual interviews with group members, lying groups demonstrated lower levels of statement-evidence consistency, within-statement consistency, and within-group consistency, compared with truth-telling groups. Consequently, if specific interview techniques are implemented during the questioning of groups then the issues associated with the consistency heuristic can be eliminated. Future studies should explore the application of other interview techniques to the interviewing of groups as well as examining the application of the SUE technique to collective interviewing contexts whereby group members are interviewed simultaneously.

The theoretical and empirical research on within-group consistency highlights both pitfalls and opportunities. On the one hand, lie catchers should be cautioned to not simply credit consistency and discredit inconsistency. On the other hand, the unanticipated question approach seems to improve the diagnostic value of the consistency cue. However, future research should explore other cues that can be measured when applying this approach to groups of interviewees (e.g., number of details or types of details). Alternative interview techniques that increase the differences between truth-telling groups and lying groups with regard to consistency need to be considered if the diagnostic value of the consistency cue is to improve.

### Memory

It is widely acknowledged that memory plays an important role in deception (Granhag and Vrij, 2005; Sporer and Schwandt, 2006; Verschuere et al., 2011; Walczyk et al., 2013). Research on memory is central to verbal veracity assessment tools, such as Reality Monitoring (RM; Johnson and Raye, 1998) and Criteria-Based Content Analysis (CBCA; Köhnken and Steller, 1988), while Bartlett’s (1932) proposition of reconstructive memory is at the heart of the ‘repeat vs. reconstruct’ hypothesis discussed above (Granhag and Strömwall, 1999). These theories approach memory from the perspective of the individual. However, cognitive psychologists have also considered memory as a social process. These social theories of memory – including collective memory (Barnier and Sutton, 2008; Rajaram, 2011) and transactive memory (Wegner, 1987; Hollingshead, 1998) – can offer unique insights into group deception. Social theories of memory can be applied to group deception in situations where multiple suspects state that they were doing something *together* at the time the crime took place.

Groups influence what individuals learn and how they remember information. Collective memory (often referred to as collaborative learning, collaborative remembering, or joint recall; Bartlett, 1932; Edwards and Middleton, 1987; Barnier and Sutton, 2008; Harris et al., 2008; Rajaram, 2011; Blumen et al., 2013) examines this social nature of memory by treating past experiences and events as memories shared with others (Barnier and Sutton, 2008; Hirst and Manier, 2008; Rajaram, 2011). Specifically, it explores how group members collectively recall information together (Rajaram and Pereira-Pasarin, 2010), and so this concept is particularly important to bear in mind when groups of interviewees are interviewed collectively about joint events.

The research investigating collective memory suggests that group collaboration can aid memory through cross-cueing (where members of the group provide cues to one another that increase recall); error-pruning (where feedback from other members of the group create discussions that make people realize their recall errors); and re-exposure (hearing other group members recall information that they themselves had forgotten; Ross et al., 2008; Blumen and Stern, 2011; Rajaram, 2011). When groups of truth-tellers are asked to recall a shared event together, they collectively recall the information, which results in truth-telling groups exhibiting interactions and communications that cannot be unveiled when they are interviewed individually (e.g., posing questions to one another, looking at one another, continuing on from one another, correcting one another, adding information to each other’s accounts, finishing each other’s sentences). Collective interviewing deception studies have shown that these interactions and communications occur more frequently for truth-telling groups than for lying groups (Driskell et al., 2012; Vrij et al., 2012; Jundi et al., 2013a,b; Vernham et al., 2014a,b). This is because the lying group members are merely recalling their planned, vague, fabricated story (Granhag et al., 2003; Strömwall et al., 2003; Vrij et al., 2010a).

The theory of transactive memory can also be applied to group deception research (Driskell et al., 2012; Jundi et al., 2013a; Vernham et al., 2014a). This theory is concerned with how groups (and individuals) process and structure information with regard to past events. It was originally developed to examine memory processes within intimate couples (Wegner, 1987), but has now been applied to various different forms of group relationships (including larger networks), such as team performance and knowledge management within the work place (Argote et al., 2003; Lee and Choi, 2003; Lewis, 2004). The theory proposes that people in close relationships share cognition and ‘think together’ by knowing each other’s memory expertise and treating one another as external memory aids (Wegner, 1987). This results in a specialized transactive memory system or ‘division of labor’ that is greater than the total of all the individual memories (Wegner et al., 1985, 1991).

The transactive memory system is active at all three stages of memory formation: encoding, storing, and retrieving. First, when information is encoded regarding a shared experience, responsibility for information is automatically divided and shared between all members of the group, so that each person knows what they are to remember as well as what the other group members are to remember (Hollingshead and Brandon, 2003). Second, when information is stored, each individual within the pair has remembering responsibilities, knowing what their role is, what they are to remember, and what information the other group members have access to Wegner et al. (1991). Third, retrieval of information is social and interactive as the group members communicate with one another to retrieve as much information as possible. The communication with one another and the discussion of incoming information enhances their individual recollections. Hollingshead (1998) refers to the ‘transaction memory search’ whereby group members who have experienced a past shared event make instinctive use of their transactive memory system to increase recall by posing questions to one another to check information or find out information, cueing one another to remind each other of further information, and handing over remembering responsibility to whoever best remembers that part of the event. These interactive and communicative behaviors between the group members help one another tap into their different memory domains and trigger further information, increasing recall.

When applied to a collective interviewing context, it has been shown that the honest groups display these fundamental interactive and communicative behaviors during joint recall significantly more than the lying groups (Vernham et al., 2014a). Lying groups, after all, are inventing shared events. Without the shared transactive memory system for encoding, storing and retrieving information, lying group members rely on their individual cognitive abilities to create a story that makes sense and matches with what the other group members are saying (Hintz, 1990). This makes it difficult for lying groups to illustrate the same degree of interactive and communicative behaviors as truth-telling groups. Consequently, deceptive communication from group members interviewed collectively is characterized by the absence of social and interactive behaviors as they recall their fabricated story (Driskell et al., 2012; Vrij et al., 2012; Jundi et al., 2013a,b; Vernham et al., 2014a,b), and only provide prepared answers to expected questions (Granhag et al., 2003; Strömwall et al., 2003; Vrij et al., 2010a).

To summarize, reconstructive memory can help explain the differences between truth-telling groups and lying groups regardless of whether the group members are interviewed individually or collectively, whereas collective memory and transactive memory can most appropriately be applied to the context of collective interviewing. Nevertheless, all three theories highlight the important role that memory plays in the recall of information and thus the detection of deception. That is, group members recalling an actual experienced joint event will do so in a different manner to group members who are attempting to recall a fabricated joint event, and the more that is understood about these differences in recall, the more that can be learnt about the possible cues to deception that may arise from groups. Future studies that explore groups within the area of investigative interviewing and deception detection should consider memory and the effects of joint recall on cues to deceit.

Although group collaboration can aid memory, it can also hinder memory. This is because other people can act as a source of misinformation whereby people conform to what other group members are saying regardless of what they themselves actually remember (Loftus, 2005). Additionally, memory contamination can occur whereby one group member causes other group members to remember information incorrectly (Gabbert et al., 2003). Whilst the memory literature suggests that collaborative groups (group members recalling information together) recall significantly more information than individual group members (each group member recalling information alone), some studies show that nominal groups (pooled individuals whereby the group members recall information individually, but details are summed so that any duplicate details are removed) recall significantly more information than collaborative groups (often referred to as *collaborative inhibition*; Basden et al., 1997; Weldon and Bellinger, 1997). Consequently, this collaborative inhibition needs to be considered when interviewing groups collectively to detect deceit.

Collaborative inhibition implies that interviewing group members separately is better than interviewing group members collectively (in terms of the amount of information obtained). However, individual interviewing of groups requires more resources and time, and is not suitable for all situations where groups need to be interviewed, for example, when there is only one interviewer available but multiple suspects (e.g., during police ‘stop and search’ or at road border control where cars containing multiple people need to be questioned). Additionally, separating the group to be interviewed removes the ability to measure communicative and interactive cues as indicators of deceit. Future research needs to consider what technique – interviewing group members individually or interviewing group members collectively – leads to the most accurate recall of information and also elicits useful, and identifiable, cues to deceit. It is likely that a combination of both individual interviewing, and collective interviewing, of group members will be required if all circumstances in which groups need to be questioned are considered. If interviewing groups individually and interviewing groups collectively are implemented into practice, future studies need to determine which interviewing technique should be implemented in which contexts, and if both techniques are needed for a particular situation, then the sequence in which they should be conducted needs to be established (i.e., interview the group collectively then individually or vice versa?).

### Group Dynamics

Before mentioning the key concepts behind group dynamics and how these may affect deception detection, we feel it important to briefly mention theories of co-offending. At least four co-offending theories have been proposed; (1) *group influence* (social learning and group pressure lead to co-offending; Akers, 1973); (2) *social selection* (offenders select each other because they share similar characteristics and interests; Feld, 1981); (3) *instrumental* (co-offending is easier, more profitable, and less risky than solo offending; Walsh, 1986); (4) *social exchange* (co-offending is a social exchange whereby offenders receive material reward, e.g., payments, and immaterial reward, e.g., social acceptance, that cannot be obtained via solo offending; Weerman, 2003).

Whilst the social exchange theory explains more of the characteristics associated with co-offending than the other three theories, none of the co-offending theories fully explain *all* the characteristics necessary to understand this type of offending. However, taken together, the theories explain: (1) why offenders choose to co-offend; (2) how co-offending takes place; (3) why there is variation between offense types in terms of the proportion of co-offending to solo offending; and (4) the instability of offending groups. An understanding of co-offending and the ways in which offenders select one another and form groups could help investigators develop more appropriate techniques for dealing with groups of offenders, particularly when it comes to establishing the best ways of interviewing these groups and determining whether they are guilty of a crime. According to social exchange theory, who is involved in a particular crime depends on which group members are available and willing (Weerman, 2003). This means that on some occasions not all group members are involved in the offense; therefore it is important to learn how different group members behave during the investigation process depending on whether they were actually involved in committing the offense (i.e., they know the specific details), or whether they are just aware of the offense (i.e., they only know who was involved, but not any details). Group members with differing knowledge about the crime will have an impact upon the amount of information that is revealed during an interview. Consequently, it is important to develop tactics that can be used to establish who knows what within a group. However, to date, no empirical studies have considered the level of knowledge distribution throughout a group and how this can affect cues to deceit.

When investigating a crime, co-offending adds a whole new aspect to detecting deceit that is not present with solo offending, *group dynamics*. Groups emerge when multiple people work together. Each of the group members bond (labeled *group formation*; Arrow et al., 2000), and as the co-offending theories illustrate, group formation is important to those offenders who are working alongside others. Groups form a structure with each member having a different role and status within the group; thus, if more can be known about how best to interview group members depending on their role or status within the group then more interview protocols can be developed to aid the detection of group deception. Unlike consistency and memory processes, group dynamics have not yet been examined in the deception detection literature, so, at present, we can only speculate about how group processes may influence deception and subsequently cues to deceit. We suggest three potential areas of group dynamics that may be relevant to deception contexts: group cohesiveness (Festinger, 1950); roles or status levels of group members (Hollander, 1985; Chemers, 2001); and cultural influences (Hofstede, 1980, 2001; Hui, 1988).

Group cohesiveness refers to the properties of a group that effectively bind the group members together to give the group a sense of solidarity (Festinger, 1950). There is suggestive evidence that groups of liars may view threats to group cohesiveness as threats to the group’s credibility. As such, groups of liars may be more concerned with maintaining an air of group cohesion compared to groups of truth-tellers. For instance, liars place weight on maintaining within-group consistency (Granhag et al., 2013), in contrast truth-tellers in collective interviewing situations may be more likely to disrupt or disagree with group members (Vrij et al., 2012). More nuanced measures of group cohesiveness could provide better cues to deceit.

The roles or levels of status that develop within a group may also be of interest. Roles facilitate group functioning, influencing how group members behave and communicate with one another. For example, those of a higher status (i.e., leaders who are deemed to be more knowledgeable and able to initiate the ideas and activities adopted by the group) will be respected more than those who are of a lower status (Hollander, 1985; Chemers, 2001). Consequently, group members who are of a lower status will be more reluctant to express disagreement with those of a higher status, but more willing to express disagreement with those who are of an equal or lower status to themselves. Communication and interaction cues are important when groups are interviewed collectively (Driskell et al., 2012; Vrij et al., 2012; Vernham et al., 2014a). By attending to group roles and the status of group members it may be possible to develop more accurate or even novel communication and interaction cues.

A final concept to consider is how cultural influences can impact group dynamics. Individualistic cultures (predominantly Western societies) highlight the importance of self-reliance, emphasizing individual needs before those of the group. Conversely, collectivistic cultures (predominantly Eastern societies) highlight the importance of interdependence, where the well-being of each individual is related to the success of the group. Emphasis is put on group loyalty and conformity, with the self-identity of each individual developing from the relationships and interconnectedness between all group members (Hofstede, 1980, 2001; Hui, 1988). The studies that have been conducted with regard to interviewing groups to detect deceit have involved participants from individualistic cultures (e.g., UK, USA, and Sweden). It is expected that findings from group deception studies using participants who support collectivism will be different from those participants who support individualism in terms of the behaviors that they show for protecting the group. It is believed that any findings obtained will be stronger from participants in collectivistic cultures because supporting the group will be more important to them, than those in individualistic cultures. Future research should address the influence of culture on group deception.

To summarize, although co-offending theories and psychological theories associated with group dynamics have not yet been applied to deception detection, they are relevant to how group members behave when being questioned. A better understanding of group dynamics will assist investigators with the best ways of interviewing group members and as a result aid with the detection of deception amongst groups.

## Future Research Ideas

The importance of studying groups (as opposed to lone individuals) is increasingly being recognized within the investigative interviewing and deception detection literature. However, until very recently, the focus was purely on interviewing group members separately (*individual interviewing*). Nowadays, studies are also being conducted into interviewing group members simultaneously (*collective interviewing*; see Vernham and Vrij, 2015 for an overview of this research). Nevertheless, there is still a long way to go before a more complete understanding of the deception occurring within groups and how to detect it is established. Future research ideas have been mentioned throughout the current review; however, there are some additional issues that should be considered if the true effectiveness of detecting deception within groups is to be recognized.

First, deception studies need to consider larger groups. At present, deception studies predominantly involve dyads, with only a few studies considering larger groups (e.g., Granhag et al., 2014 used triads and Sooniste et al., 2014 used quartets). It should be possible to apply the findings obtained from the already completed deception experiments using groups to future studies with more than two interviewees, because the theoretical rationale on which the already obtained findings are based (e.g., consistency heuristic, reconstructive memory, transactive memory) should remain the same regardless of group size. However, as group size increases, it is likely that more cues to deceit will be elicited, because the interview process will become more challenging (particularly for liars) as each group member will have more people to manage and correspond with.

Second, there are a number of group processes that could be explored in addition to the ones already mentioned. For example, it would be interesting to consider what would happen if individual group members do not know whether their fellow group members are also being interviewed – how does this affect their choice of strategy and the information they disclose? The ‘Prisoner’s Dilemma’ is similar, but the group members would instead need to make decisions about cooperating with the group when lacking the knowledge about which group members are actually being interviewed. Additionally, the order in which each group member thinks they are being interviewed could be strategically used during an interview. For example, does the amount of information produced and the elicitation of cues to deceit depend on whether the group member believes they are first, or last, to be questioned? Another idea for a further study would be to include an additional dependent variable where each group member is asked who else they think is being questioned and what information they think their fellow group members will provide.

Third, deception detection studies tend to compare truth-telling participants with lying participants who have perpetrated some kind of misdemeanor that is engineered by the researchers. In real-life, it is not necessarily this clear-cut, especially if a guilty group of suspects has completely innocent people within it. Future research should explore how mixed groups of innocent and guilty individuals behave when interviewed either individually or collectively, and what cues to deceit emerge.

Fourth, the current review has considered each of the theories associated with group deceit as if they are independent of one another. However, it would be interesting for future studies to not only consider the application of each of the individual theories to group deceit and its detection, but also to consider the application of a combination of the theories to group deceit and its detection. For example, the link between joint memory recall and group dynamics and the effect this has on the elicitation of cues to deceit.

Fifth, the vast majority of studies have focused on co-offenders when other group situations are relevant for law enforcement. To our knowledge, the study by Nahari and Vrij (2014) examining deception in alibi witness situations, is the only group deception study to consider groups other than co-offenders. For a complete understanding of group deception more research of this kind is essential. Additionally, research exploring the best way of determining the accuracy of information provided when multiple witnesses are questioned about the same crime/event would contribute to the knowledge-base on the elicitation of information from groups.

Finally, studies examining the individual interviewing of groups have focused primarily on consistency as a cue to deceit, whereas collective interviewing studies have focused primarily on social cues to deceit. Future studies should therefore measure alternative cues when group members are interviewed individually and/or collectively in order to determine what other cues to deceit can be elicited from groups to enable the correct classification of groups based on veracity.

## Practical Implications

Over recent years research has increased our knowledge about what is happening during suspect interviews (e.g., Soukara et al., 2009; Walsh and Bull, 2010). However, in order to inform practice, there is a need to reach beyond descriptive research that focuses on gathering facts, and set up studies that can generate more normative knowledge. By doing such research one can identify how the current approaches that are used during investigative interviewing can be improved, which can also help to identify which approaches are most effective in each given context.

Although some law enforcement personnel currently conduct collective interviews in some situations, the interview manuals and training programs typically utilized by police and other law enforcement agencies focus on the interviewing of lone individuals. Consequently, the interviewing tactics and techniques that investigators are primarily taught, such as the PEACE model (ACPO, 2001) or Behavioral Analysis Interview (BAI; Inbau et al., 2001), and the cues to deceit that they choose to measure (e.g., consistency or nervousness; Vrij, 2008), are developed around research into lone individuals. Hence, these interview manuals and training programs have very little, if any, information about how best to interview groups and what deception cues to observe when more than one individual is being interviewed about a joint offense. As highlighted within the current review, knowledge about lone individuals cannot always translate to groups of individuals, yet co-offending occurs frequently. The current review demonstrates that by not truly understanding groups, a large number of opportunities to employ novel or existing interviewing techniques to detect deceit are being missed (e.g., the ability to apply a collective interviewing approach or how best to measure and elicit within-group consistency). Hence, by offering both theoretical and empirical insights on how to interview groups of suspects, the current review can inform policy and practice. It suggests that the framework of police interview manuals and training programs should be revised to include more specific tactics and techniques for group situations. If this occurs then investigators will have a wider range of tools and a greater understanding of what interviewing techniques and tactics to employ when they have a co-offending situation and need to detect deception occurring within groups.

Furthermore, the current review has implications that stretch beyond the traditional law enforcement context. For example, it might guide policy with respect to techniques for both collecting and assessing the reliability of human intelligence. Specifically, the recent congressional amendment—To Reaffirm the Prohibition on Torture Amendment (2015)—strictly limits the US interrogation procedures to the methods listed in the (US Army Field Manual 2-22.3 (FM 34-52), 2006). The amendment also requires the regular update of the manual based on the best available scientific evidence. Currently, the Army Field Manual says little about group interviewing situations. The current review therefore acts as a first step to fill this gap and as a call for researchers to continue important work on this topic area.

## Conclusion

The interviewing of groups to detect deceit about joint events is different from interviewing lone individuals to detect deceit about solo events. In group situations, not only can group deception be explored but so can individual deception, resulting in the measurement of considerably more cues to deceit. This is because unique cues that cannot be explored in lone individuals can also be measured, such as within-group consistency or cues stemming from the way that group members communicate and interact with one another. Additionally, being part of a group is more cognitively demanding in itself because each group member has to think about what they say as well as what others might say. Whether group members should be interviewed individually or collectively depends on each given situation. As the current review highlights, there are benefits to both techniques and unique cues to deception that can emerge depending on the interview style employed. Overall, there is the opportunity for investigators to develop interview protocols based on group dynamics that allows for key differences between truth-telling groups and lying groups to emerge. There is currently a lack of studies exploring group deception and its detection, yet a clearer and more accurate understanding of the deception occurring within groups and the strategies groups employ would benefit criminal, security, and intelligence investigations; and thus be of value to crime prevention and policy.

## Author Contributions

All three authors worked together to develop the idea about the review paper and how it should be structured. ZV did the majority of the writing and put the manuscript together. ZV received frequent comments and amendments from both P-AG and EM throughout the writing process. Several meetings were held with all three authors present. The manuscript has been checked by all three authors prior to submitting.

## Conflict of Interest Statement

The authors declare that the research was conducted in the absence of any commercial or financial relationships that could be construed as a potential conflict of interest.
