# Corrigendum: Detecting Deception within Small Groups: A Literature Review

**DOI:** 10.3389/fpsyg.2016.01160

**Published:** 2016-08-02

**Authors:** Zarah Vernham, Pär-Anders Granhag, Erik Mac Giolla

**Affiliations:** ^1^Department of Psychology, University of PortsmouthPortsmouth, UK; ^2^Department of Psychology, University of GothenburgGothenburg, Sweden; ^3^Norwegian Police University CollegeOslo, Norway; ^4^Department of Psychology, University of OsloOslo, Norway

**Keywords:** group deception, interviewing, strategies, consistency, memory

In the original Review article, the third author's name was published as “Erik M. Giolla”. His name should be Erik Mac Giolla. The author list has been corrected above.

The authors apologize greatly for this mistake. This error does not affect the scientific conclusions of the article in any way.

The original article has been updated.

## Author contributions

All three authors worked together to develop the idea about the review paper and how it should be structured. ZV did the majority of the writing and put the manuscript together. ZV received frequent comments and amendments from both PG and EM throughout the writing process. Several meetings were held with all three authors present. The manuscript has been checked by all three authors prior to submitting.

### Conflict of interest statement

The authors declare that the research was conducted in the absence of any commercial or financial relationships that could be construed as a potential conflict of interest.

